# Low Levels of Amino Acid Auxotrophy Among Environmental *Pseudomonas* Isolates

**DOI:** 10.1111/1462-2920.70434

**Published:** 2026-10-02

**Authors:** Simon Maréchal, Benjamin Heiniger, Shaohua Gu, Swagatika Dash, Christian H. Ahrens, Rolf Kümmerli

**Affiliations:** ^1^ Department of Quantitative Biomedicine University of Zurich Zurich Switzerland; ^2^ Method Development and Analytics Agroscope Zurich Switzerland; ^3^ PhD Program Systems Biology Zurich Life Sciences Graduate School Zurich Switzerland; ^4^ College of Resources and Environmental Sciences, State Key Laboratory of Nutrient Use and Management (SKL‐NUM), National Academy of Agriculture Green Development, China Agricultural University Beijing China; ^5^ Department of Ecology, School of Biology/Chemistry University of Osnabrück Osnabrück Germany; ^6^ Swiss Institute of Bioinformatics Zurich Switzerland

**Keywords:** alternative biosynthetic pathways, amino acids auxotrophy, bioinformatic prediction, natural bacterial communities, phylogenetic associations, soil and pond habitats

## Abstract

Auxotrophy, the inability of bacteria to synthesize one or multiple essential metabolites, is thought to be common among bacteria. However, studies often rely on either bioinformatic genome‐based prediction of auxotrophies or on experiments with low strain numbers. Here, we combine experimental and bioinformatic approaches to assess amino acid auxotrophies among 315 co‐isolated natural *Pseudomonas* strains from pond and soil habitats. Experiments revealed that *Pseudomonas* isolates are predominantly prototrophs. We identified one histidine auxotroph, one auxotroph with complex intertwined dependencies and four ‘fragile’ phenotypes showing delayed growth without supplemented amino acids. Applying three bioinformatic pipelines largely confirmed experimental data but yielded specific biases in auxotrophy over‐ or underestimation. Moreover, none of the pipelines could resolve the genetic basis of the slow‐growing phenotypes or of more complex dependencies. Our analysis further revealed the existence of multiple alternative biosynthesis pathways for methionine, proline and phenylalanine, with significant pathway enrichments being linked to phylogeny and habitat. We conclude that combining experiments with bioinformatics is a powerful approach to assess the metabolic potential of environmental bacteria. Moreover, taxa like *Pseudomonas* can be predominantly prototrophic possibly owing to their generalist lifestyle, thus calling for nuanced ecological concepts predicting auxotrophy levels based on lifestyle and habitat.

## Graphical abstract

We explored the frequency of auxotrophy among co‐isolated natural *Pseudomonas* spp. We used a combination of in vitro and in silico approaches to assess the frequency of auxotrophy in communities and to understand the association between habitat, phylogeny and the occurrence of specific amino acid metabolic pathways. Our results show that natural *Pseudomonas* spp. are mainly prototrophic. Created in BioRender. Kuemmerli, R. (2026) https://BioRender.com/h1my1d7.

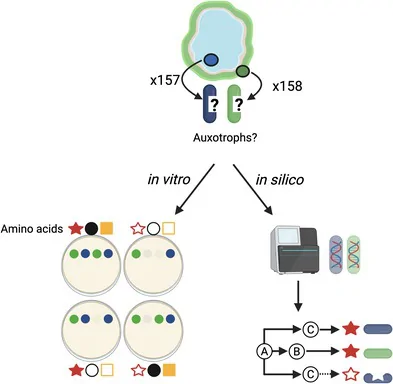

## 1 Introduction

Most environments are populated by complex bacterial communities consisting of many different species (Gibbons and Gilbert 2015; Sunagawa et al. 2015). Given that space and nutrients within these communities are typically limited, there are ample opportunities for bacteria to interact in either negative or positive ways (Foster and Bell 2012; Ghoul and Mitri 2016; Kehe et al. 2021; Figueiredo et al. 2022). Negative interactions are ubiquitous and typically involve indirect competition for resources or direct interference competition through various mechanisms including the release of toxins or the deployment of contact‐dependent inhibition systems (Ghoul and Mitri 2016; Granato et al. 2019). Positive interactions also occur and often manifest in the release of beneficial products and metabolites that can be used by other community members (West et al. 2007; Kost et al. 2023). Such cooperative interactions can be based on the sharing of extracellular enzymes, siderophores and biofilm components (Nadell et al. 2009; Garcia‐Garcera and Rocha 2020; Kramer, Özkaya, et al. 2020; Pontrelli et al. 2022), or can involve metabolic interactions (Schink 2002; Morris et al. 2012; D'Souza et al. 2018; Kehe et al. 2021; Giri et al. 2022; Pauli et al. 2023; Hesse and O'Brien 2024).

The basic premise of metabolic interactions is that not all bacterial species in a community need to encode all the essential metabolic pathways. Instead, different bacteria can specialize on specific pathways or pathway steps and release metabolites into the environment, which can then be used as nutrient sources by metabolically complementary species in the community (Johnson et al. 2012). Such metabolic interactions can foster dependencies between species as community members may lose specific pathways altogether and thus shift from an autonomous (prototrophic) to a dependent (auxotrophic) lifestyle (Morris et al. 2012; Seif et al. 2020). Dependencies can either be linear, whereby species B relies on the release of a metabolite from species A, or circular, whereby species A and B mutually depend on the release of specific metabolites from the other species (Harcombe et al. 2018). Metabolic interactions can promote evolutionary genome reduction and streamlining, whereby species lose one or several biosynthetic pathways and thereby become fully dependent on the use of external sources of the corresponding metabolites (Wolf and Koonin 2013).

Studying metabolic interactions and the evolution of dependencies has become popular in recent years (D'Souza et al. 2014; Pande and Kost 2017; Johnson et al. 2020; Kim et al. 2021; Starke et al. 2023; Kasalo et al. 2025). This is because metabolic interactions are regarded as key elements explaining microbial community diversity and stability, and in the context of hosts they have been associated with host health and disease (Ramsey et al. 2011; Goldford et al. 2018; Starke et al. 2023). However, current study approaches come with specific limitations. Large‐scale metabolic interaction studies are typically based on bioinformatic modelling (Price et al. 2020; Seif et al. 2020; Zimmermann et al. 2021). Although these approaches are powerful to predict metabolic interactions in complex communities, experimental validation is often not possible. Conversely, empirical studies provide solid experimental data but are often restricted to a relatively low number of (culturable) species or strains (Harcombe et al. 2018; Giri et al. 2021). Consequently, we still have a limited understanding of how common auxotrophies and metabolic dependencies are among strains and species in natural communities, and how good the match between bioinformatic approaches and empirical data is.

In our work, we aim to address these two open questions by connecting empirical experiments with bioinformatic analysis. Specifically, we take advantage of a well‐characterized collection of 315 *Pseudomonas* isolates sampled from two habitats (soil and pond) (Butaitė et al. 2018; Kramer, López Carrasco, et al. 2020) to examine the ability of the isolates to synthesize proteinogenic amino acids. First, we assessed the frequency and specificity of amino acid auxotrophy using culture‐based approaches. Second, we used different bioinformatic tools to screen the genomes of all isolates to detect amino acid biosynthetic pathways, the overall diversity of pathways, and potential gaps therein. Third, we compared experimental with bioinformatic data to examine the level of consistency across the approaches. Finally, we tested whether the abundance of specific amino acid pathways correlates with habitat type (soil vs. pond) or phylogeny of the isolates.

## 2 Results

### 2.1 Natural *Pseudomonas* Isolates Show Low Levels of Amino Acid Auxotrophy

In a first assay, we grew each of the 315 strains for 24 h in either M9 minimal medium with glucose (M9G) but without amino acids or in M9G supplemented with casein hydrolysate (a source of proteinogenic amino acids) both on solid agar (Figure 1A) and in liquid shaken culture (Figure S1). We expect prototrophs to grow well in both media and under both culturing conditions, whereas auxotrophs should only be able to grow in the presence of supplemented amino acids (Figure 1B). When qualitatively comparing the growth of colonies on agar across the two media, we found that 92.4% (291 of 315) of all isolates grew well in the absence of supplemented amino acids, suggesting that these isolates are amino acid prototrophs (Figure 1C). Among the remaining 24 isolates, five isolates displayed a complete absence of growth whereas 19 isolates showed reduced growth in amino acid depleted medium compared to the medium with amino acids (Figure 1C). We obtained highly congruent results for the liquid shaken conditions for most isolates (Figure S1). However, there were also a few isolates with condition‐specific growth preferences, particularly one isolate that did not grow on agar yet grew well in liquid culture and six isolates that grew well on agar but showed poor growth in liquid culture. Taken together, the results from our first screen suggest that most of our natural *Pseudomonas* isolates are amino acid prototrophs.

**FIGURE 1:**
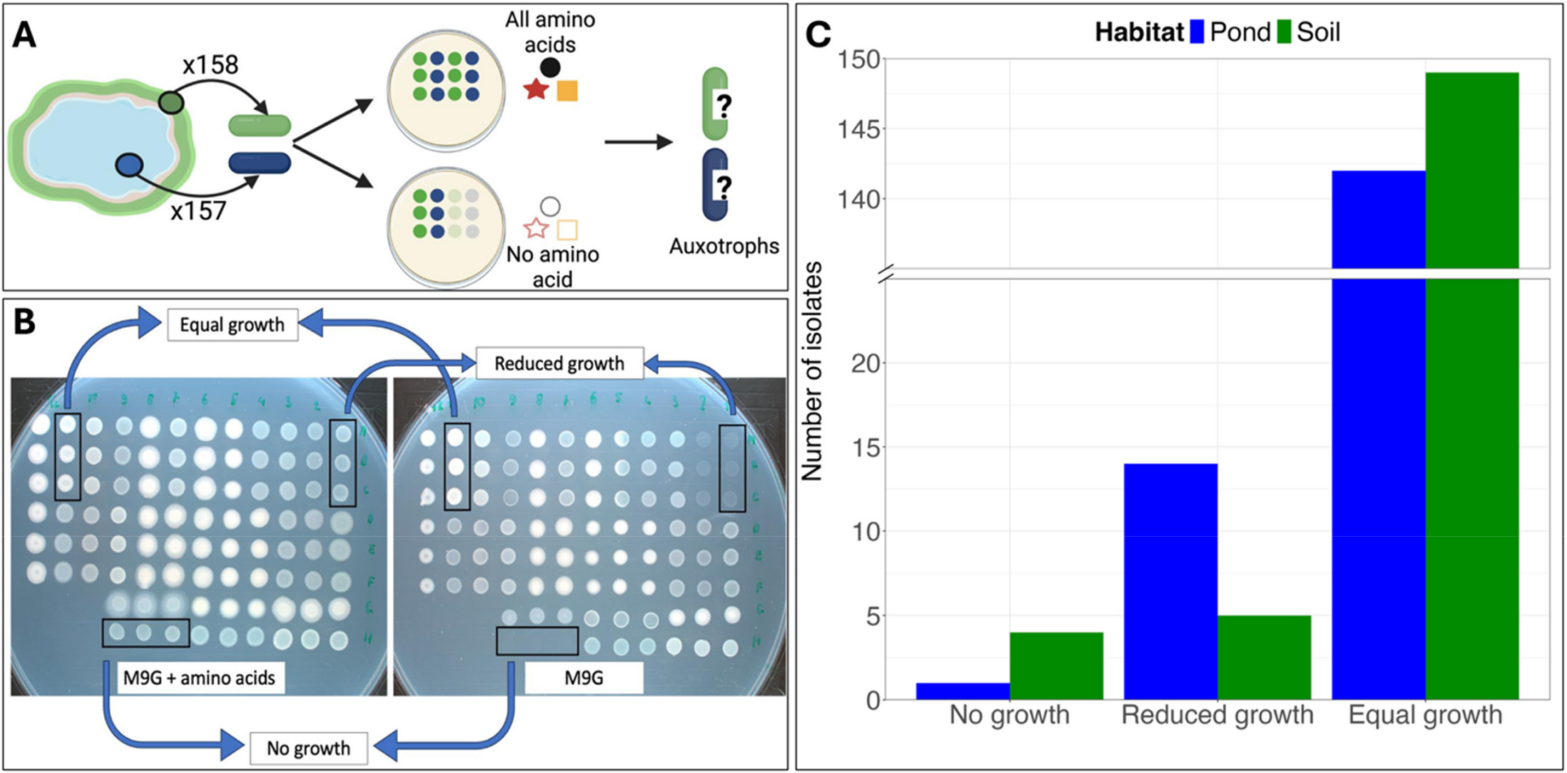
Amino acid growth assays revealed low levels of auxotrophy among natural *Pseudomonas* isolates: (A) 315 natural *Pseudomonas* isolates, originally sampled from pond and soil habitats, were grown on M9G containing either all or none of the proteinogenic amino acids to detect putative amino acid auxotrophies. See Butaitė et al. (2021) for a description of the sampling scheme and note that soil samples were collected from a meadow that is physically separated from the pond. (B) Representative experimental plates showing the growth of the same set of environmental isolates on the two different M9G media in triplicates. Growth between media was compared qualitatively and categorized as follows: ‘equal growth’ when no growth difference was observed between the two media, ‘reduced growth’ when the growth was reduced on the medium without amino acids and ‘no growth’ when no detectable growth could be observed on the medium without amino acids. Growth phenotypes were highly consistent across the three replicates and time (24 h vs. 48 h). (C) Number of isolates in each growth category split according to habitats (blue = pond, green = soil). Figure 1A created in BioRender. Kuemmerli, R. (2026) https://BioRender.com/h1my1d7.

### 2.2 Experiments in Defined Media Identified Two Isolates With Amino Acid Auxotrophies

In a second assay, we used a defined M9G medium by individually adding 50 μM of each of the 20 proteinogenic amino acids (see Figure S2 for data on minimal amino acid requirements). We used the high‐throughput agar assay to rescreen the growth of the 24 isolates initially displaying reduced or no growth in the absence of amino acids under these defined conditions for both 24 h and 48 h. This assay revealed that 18 out of 24 isolates showed consistent growth with or without supplemented amino acids, suggesting that they are prototrophs (Figure 2A). Moreover, we found four isolates with equal growth in both media but with the growth recovery only becoming visible after 48 h in medium without amino acids. Finally, we identified two isolates (s3h05, p3F08) displaying a clear absence of growth in medium without amino acids even after 48 h. In summary, our assay in defined medium identified two auxotrophic isolates and four isolates with very slow growth in the absence of supplemented amino acids (Figure 2A).

**FIGURE 2:**
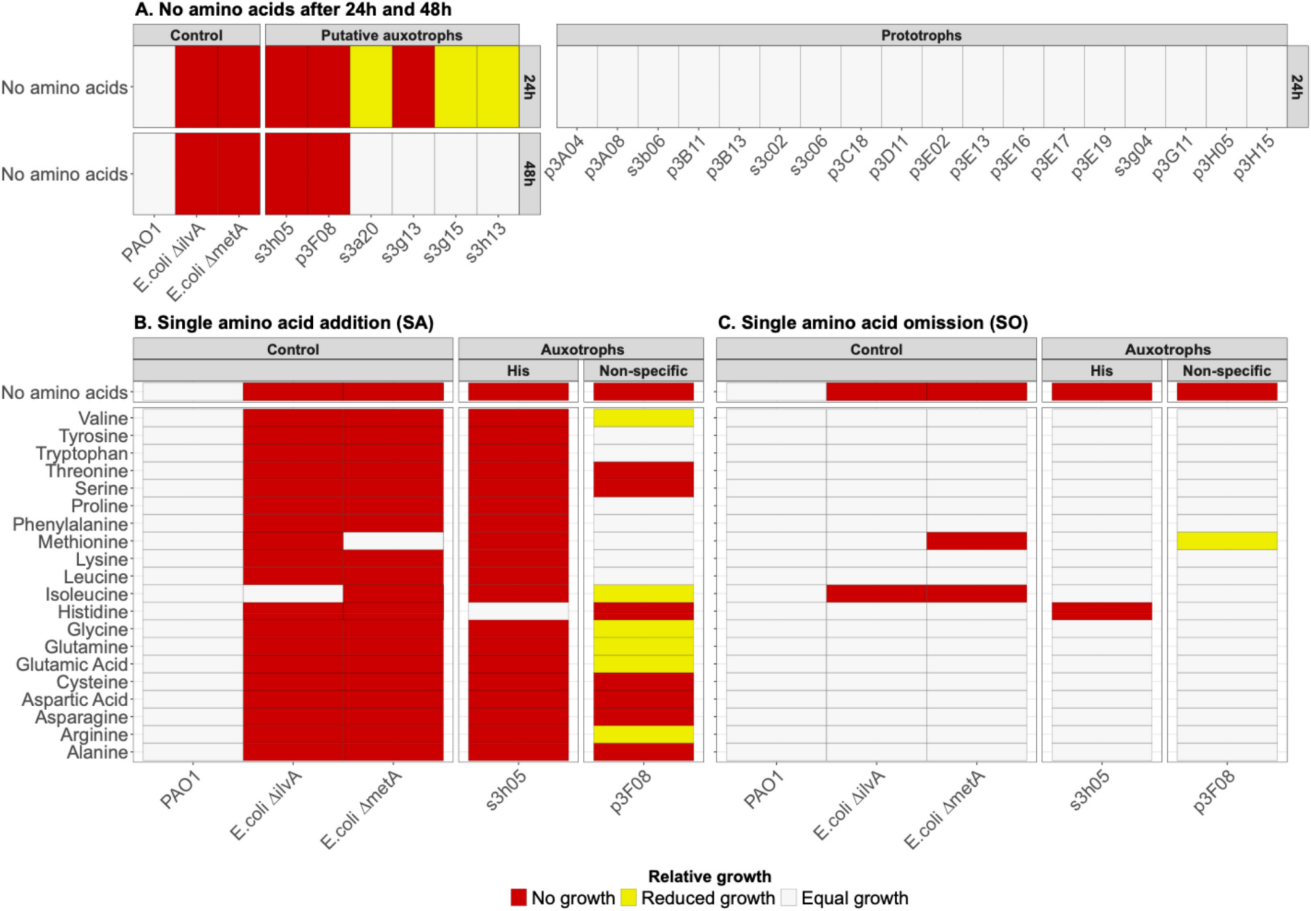
In depth growth assays of non‐ and slow‐growing isolates revealed a single specific and a single non‐specific auxotroph: (A) The 24 *Pseudomonas* isolates that showed no or reduced growth in the initial screen (Figure 1) were grown on M9G agar medium supplemented with either 50 μM of each amino acid or no amino acids. Their relative growth values were assessed as described in Figure 1B after 24 h and also after 48 h for isolates displaying reduced growth after 24 h. This assay revealed 18 isolates to be prototrophs, four isolates to be metabolically ‘fragile’ slow growers and two isolates to be auxotrophs. (B) Single amino acid addition (SA) and (C) single amino acid omission (SO) experiments to assess the auxotrophy of isolates s3h05 and p3F08, including three control strains: *P. aeruginosa* PAO1 (prototroph) and *E. coli* Δ*ilvA* and *E. coli* Δ*metA* (auxotrophs). The specific amino acid added to or omitted from M9G are shown on the *y*‐axis. Growth levels are expressed relative to the growth of each isolate in medium containing all amino acids.

To specify the precise amino acid auxotrophy in isolates s3h05 and p3F08, we designed two additional assays in which each isolate was cultured in M9G medium supplemented with either one single amino acid (single addition, SA, Figure 2B) or omitting a single amino acid from the mix of 20 (single omission, SO, Figure 2C). This experimental design allowed us to distinctively identify specific amino acid auxotrophies. For instance, a tryptophan auxotroph would grow in M9G supplemented with tryptophan as the sole amino acid source (SA) but would not grow in M9G supplemented with 19 amino acids lacking tryptophan (SO). To validate our approach, we used a subset of 12 *Escherichia coli* mutants from the KEIO collection (Mee et al. 2014) where each mutant was deficient for the synthesis of a single specific amino acid. When growing these mutants in M9G SO media, we found that all 12 *E. coli* mutants failed to grow in the medium lacking the respective amino acid, as expected (Figure S3A,B). Notably, all these mutants also failed to grow in M9G without isoleucine, a pattern that can be explained by a phenomenon called ‘valine‐induced isoleucine starvation’, which is common among *E. coli* strains (Gummesson et al. 2020), see Figure S3C,D for detailed experiments on this phenomenon. Overall, these control experiments demonstrate that our approach worked and showed that single amino acid auxotrophies can reliably be identified with our SA/SO approach. With this prerequisite established, we conducted the SA and SO experiments with isolates s3h05 and p3F08 (Figure 2B,C). We found strong evidence for a specific histidine auxotrophy in isolate s3h05, whereas isolate p3F08 displayed patchy growth vs. non‐growth patterns with SA, but could always grow with SO. This result suggests a more intricate metabolic dependency, henceforth called non‐specific auxotrophy.

### 2.3 Growth Phenotype of p3F08 Is Explained by a Combined Proline, Methionine, Cysteine Dependency

Since the growth pattern of isolate p3F08 is incompatible with both prototrophy and specific auxotrophy, we suspected a complex metabolic dependency. Such complex dependencies could be common among bacteria but may easily be overlooked in simple screening assays. This motivated us to investigate this isolate in more detail. First, we realized that qualitative growth assessment on agar plates was ambiguous for this isolate because of the formation of a visible ring at the droplet edge (Figure S4). Because it is unclear whether this ring represents growth, we moved towards a quantitative assessment of growth using Fiji (Figure S5) and repeated the SA and SO experiments. Second, it is well established that the synthesis pathways for several amino acids can be interconnected, which we suspected to be the source for the complex phenotype observed in p3F08. We thus conducted dual and triple amino acid addition and omission experiments for amino acids known to have interconnected pathways in *P. aeruginosa* : (1) isoleucine, leucine and valine pathways interconnected via the enzyme *IlvC* and (2) methionine, cysteine and serine pathways sharing similar intermediates that could be used to compensate for a specific amino acid auxotrophy.

The SA experiments revealed clear evidence for both a proline and a methionine auxotrophy in p3F08 (Figure 3). However, this isolate grew perfectly fine in the SO experiments when one of the two amino acids was omitted, again confirming that the auxotrophies can potentially be compensated through interconnected pathways. Indeed, the dual and triple amino acid omission experiments revealed that the methionine auxotrophy was masked in the presence of cysteine but became apparent as a slow‐growing phenotype when both methionine and cysteine were lacking. We conclude that alteration in interconnected pathways can lead to complex phenotypes and metabolic dependencies that are easily missed in simple metabolite absence/presence screens.

**FIGURE 3:**
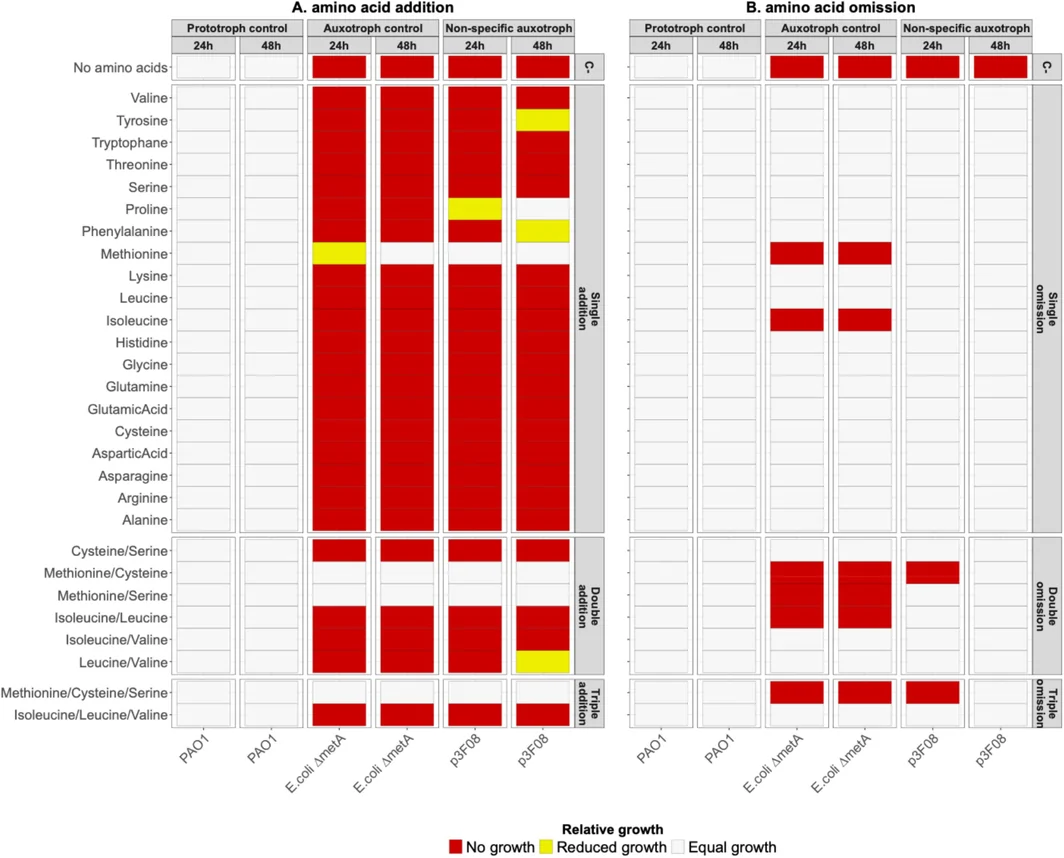
The complex phenotype of isolate p3F08 is explained by a proline and an interlinked methionine–cysteine dependency: Isolate p3F08 was subjected to single amino acid addition (SA) and single amino acid omission (SO) treatments along with a set of double and triple amino acid additions and omissions to elucidate the complex amino acid dependencies in this isolate. For the double and triple amino acid experiments, we considered two biosynthetically connected triplets: Methionine/cysteine/serine and isoleucine/leucine/valine. Controls include *P. aeruginosa* PAO1 (prototroph) and *E. coli* Δ*ilvA* and *E. coli* Δ*metA* (auxotrophs). To rule out ambiguity associated with qualitative growth assessment, we switched to quantitative Fiji‐based growth measurements. Growth is expressed relative to the medium with all amino acids. The specific amino acid(s) added to or omitted from M9G are shown on the *y*‐axis.

### 2.4 Bioinformatic Analysis Confirm That Most *Pseudomonas* Isolates Are Prototrophs

To complement our experimental data, we conducted bioinformatic analyses on the sequenced genomes of 314 *Pseudomonas* natural isolates (one isolate was excluded from the analysis due to poor sequencing quality). We aimed to (i) create an inventory of all amino acid synthesis pathways present in each isolate, (ii) identify incomplete pathways that could explain the observed auxotrophies and (iii) check for the presence of alternative pathways for the same amino acid across isolates. For these analyses, we used GapMind (Price et al. 2020), a software that can predict biosynthetic pathways of 17 amino acids and putative gaps therein with a confidence score (Figures 4 and S6). Notably, GapMind cannot predict biosynthetic pathways for alanine, aspartate and glutamate. This is because these pathways involve intermediates from central metabolism and non‐specific transaminases that are generally present in genomes. Thus, it is generally assumed that bacteria can produce these three amino acids (Price et al. 2020). As prediction confidence can vary depending on the annotation method used, we ran GapMind on our genomes annotated with three different pipelines: Prokka, NCBI Prokaryotic Genome Annotation Pipeline (PGAP) and NCBI ORFfinder (ORFfind).

**FIGURE 4:**
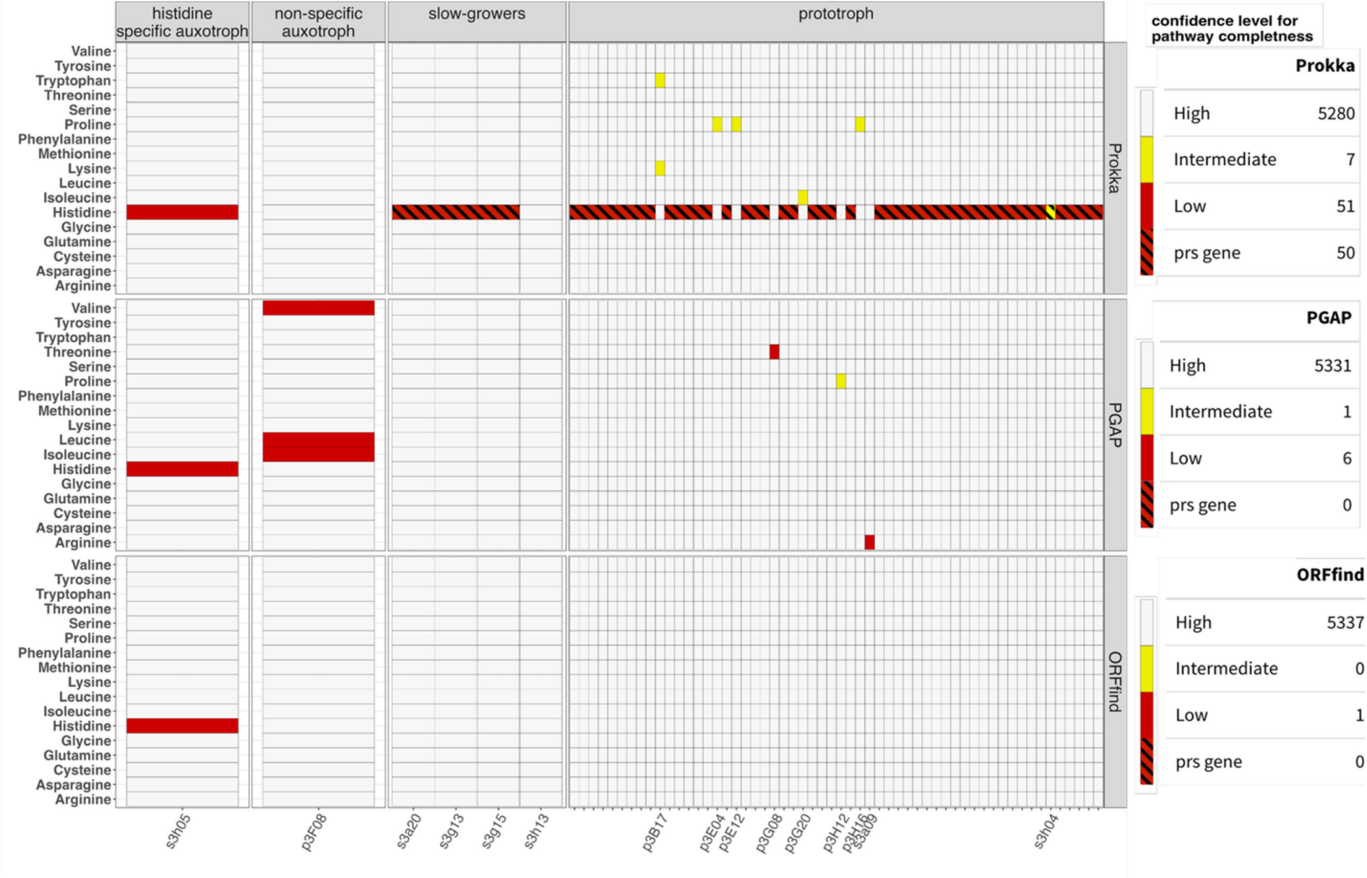
Comparing three bioinformatic pipelines to predict gaps in amino acid synthesis pathways: GapMind was used to predict amino acid synthesis pathways and possible gaps therein, from whole genome sequence data of 314 natural *Pseudomonas* isolates. The plot shows predicted gaps based on three different annotation pipelines (top row = Prokka, middle row = PGAP, bottom row = ORFfind). Isolates are ordered based on their experimentally determined amino acid phenotypes (from left to right: Histidine‐specific auxotroph, non‐specific auxotroph, fragile slow growers, prototrophs). Isolates with complete predicted pathways for all amino acids are not shown in this figure but can be found in Figure S6A–C. For prototrophs, only the isolate with a predicted gap other than the *prs* gene (red‐black hatched boxes) are named. Tables on the right show the numbers of predicted amino acid synthesis pathways across all 314 isolates, stratified by annotation pipeline and prediction confidence: High (light grey), intermediate (yellow) and low (red). Low confidence predictions for *prs* (red‐hatched) are shown separately. A critical point is that the confidence levels refer to the presence of a gene, such that low confidence for a pathway stands for high confidence for a gap.

All three pipelines confirmed that most *Pseudomonas* isolates are prototrophs. Specifically, we detected 5280, 5331 and 5337 complete amino acid synthesis pathways with high confidence among the 5338 total expected hits (314 isolates × 17 proteinogenic pathways) with Prokka, PGAP and ORFfind, respectively (Figure 4). Accordingly, putative gaps were rare. Prokka predicted 58 gaps, whereas PGAP and ORFfind predicted only seven and one gaps, respectively. Only one gap was consistently predicted across the three annotation pipelines. It concerned a single gene (*hisF*), which encodes an imidazole glycerol phosphate synthase involved in histidine synthesis (Figure S6B). The isolate s3h05 that was predicted to lack *hisF* was indeed the specific histidine auxotroph identified in our experiments, thus yielding a perfect match between experimental and bioinformatic data.

Annotation with Prokka returned 50 putative gaps in the gene *prs* encoding a ribose phosphate pyrophosphokinase involved in the histidine biosynthesis pathway (Figure S6B). Since these gaps were not predicted by ORFfind and PGAP, we suspected them to be false positives. Indeed, all isolates with a predicted *prs* gap were prototrophs in our experiments. Moreover, the inflation in *prs* gap predictions with Prokka is a known problem for *Pseudomonas fluorescens* strains (Price et al. 2020).

With the PGAP annotation as basis, GapMind predicted five candidate gaps (in addition to *hisF*). One gap each was predicted for the arginine (isolate s3a09) and the threonine (isolate p3G08) pathways. We considered them false positives because these predictions were only supported by one annotation method and moreover concerned isolates that had a prototrophic phenotype. The three remaining gaps all concerned the isolate p3F08 (the non‐specific auxotroph with complex dependencies, Figure 3) and a single gene (*ilvC*) encoding an enzyme connecting the synthesis pathways of isoleucine, leucine and valine. However, we must also consider these hits as false positives because experimental data do not support any evidence for auxotrophies associated with these interconnected pathways.

In summary, our bioinformatic analyses reinforced the observed high levels of prototrophy among environmental *Pseudomonas* isolates and confirmed the only experimentally identified single amino acid auxotroph. In addition, we found that bioinformatic pipelines differ in their extent to which they generate false positive predictions and they were unable to unravel the mechanistic basis of the non‐specific auxotroph p3F08.

### 2.5 GapMind Reveals Alternative Pathways for Methionine, Proline and Phenylalanine Biosynthesis

We further used GapMind to detect alternative amino acid biosynthesis pathways encoded among our *Pseudomonas* isolates. The rationale for this analysis is to capture the metabolic diversity among pseudomonads. We found alternative pathways for methionine, proline and phenylalanine biosynthesis (Figures 5 and S6B,C).

**FIGURE 5:**
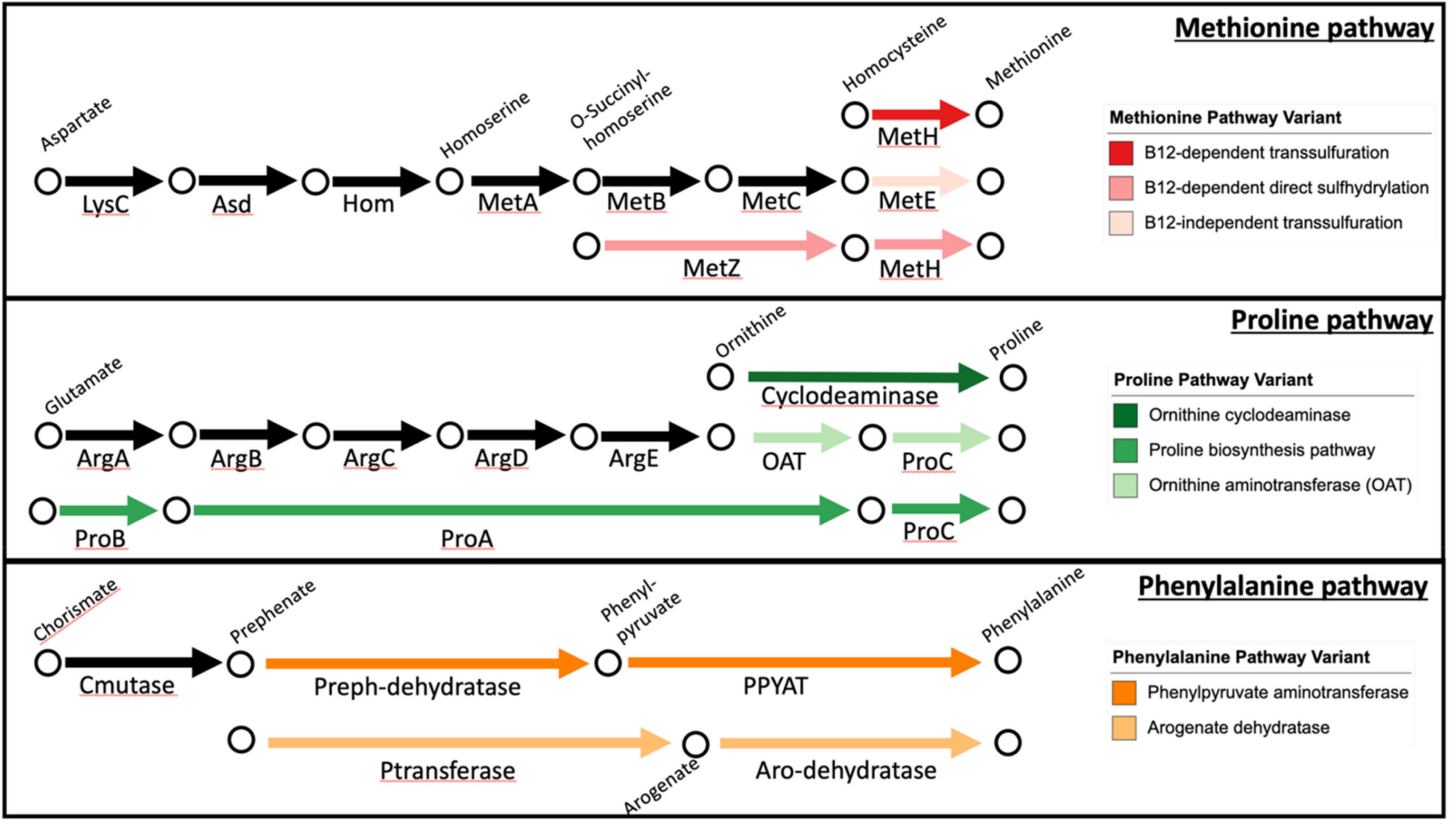
Alternative metabolic pathways for methionine, proline and phenylalanine. Shown are the alternative pathways for methionine, proline, phenylalanine biosynthesis encoded among the 314 natural *Pseudomonas* isolates. Whereas two strains can differ in the pathway they possess, none of the strains featured two alternative pathways for the same amino acid. Black arrows represent steps that are shared among at least two pathways, whereas coloured arrows show steps unique to each pathway. Biosynthetic pathways were reconstructed using KEGG and BioCyc database.

For methionine, the first four biosynthetic steps are conserved before the pathway splits into two branches, whereby O‐succinyl‐homoserine is converted to homocysteine either via the transsulfuration pathway (sulphur source: cysteine, enzymes: MetB and MetC) or the direct sulfhydrylation pathway (sulphur source: H_2_S; enzymes: MetZ). A second branching occurs in the transsulfuration pathway, in which homocysteine can be converted to methionine either via a vitamin B12‐dependent (enzyme: MetH) or B12‐independent (enzyme: MetE) pathway. We found that the B12‐dependent transsulfuration was the most common pathway among the natural *Pseudomonas* isolates with a total of 278 occurrences (pond: 125, soil: 153, Table 1). The B12‐dependent sulfhydrylation was the second most common pathway with 35 occurrences (pond: 31, soil: 4). Finally, we found only one occurrence of the B12‐independent transsulfuration.

**TABLE 1:** Distributions of alternative amino acid biosynthetic pathways across soil and pond habitats and their association with phylogeny.

Amino acid	Pathway variant	Number of variant		Association between pathways and habitats		Association between pathways and phylogeny		
		In pond	In soil	*p*‐Values of overall pathway distribution^a^	*p*‐Values of post hoc pairwise comparisons^b^	Fritz & Purvis' *D* statistic	*p*‐Values for random phylogenetic structure^c^	*p*‐Values from pGLMM^d^
Methionine	B12‐dependent transsulfuration	278		< 0.0001	< 0.0001	0.0280	< 0.0001	0.1244
		125 (45%)	153 (55%)					
	B12‐dependent direct sulfhydrylation	35			< 0.0001	−0.0042	< 0.0001	0.0907
		31 (88.6%)	4 (11.4%)					
	B12 independent transsulfuration	1			/	/	/	/
		0 (0%)	1 (100%)					
Proline	Ornithine cyclodeaminsae	169		0.0065	0.4230	0.0228	< 0.0001	0.2233
		77 (45.6%)	92 (54.4%)					
	Proline biosynthesis pathway	137			1.0000	0.0976	< 0.0001	0.6231
		71 (51.8%)	66 (48.9%)					
	Ornithine aminotransferase	8			0.0102	0.0329	< 0.0001	/
		8 (100%)	0 (0%)					
Phenylalanine	Phenylpyruvate aminotransferase	238		< 0.0001	/	0.1983	< 0.0001	0.0097
		103 (43.3%)	135 (56.7%)					
	Arogenate dehydratase	76			/	0.1982	< 0.0001	0.0097
		53 (69.7%)	23 (30.3%)					

^a^ Statistics based on Fisher's exact test on 2 × 3 contingency table with 10′000 Monte Carlo simulations.

^b^ Statistics based on Fisher's exact test on 2 × 2 contingency table, Bonferroni corrected for multiple testing.

^c^ Statistics based on Fritz & Purvis' *D*: null hypothesis (absence of phylogenetic signal): *D* = 1; alternative hypothesis (phylogenetic signal): *D* = 0.

^d^ Statistics based on a phylogenetic generalized linear mixed model (pglmm) testing for habitat enrichment of alternative pathways while controlling for phylogenetic associations.

For proline, there were two completely distinct pathways. The first biosynthetic pathway represents the standard proline synthesis route consisting of three enzymes (ProA–C). It was present in 137 isolates (pond: 71, soil: 66). The second biosynthetic pathway builds on the arginine pathway (enzymes: ArgA–E), which first yields ornithine. Ornithine is then converted to proline through either a cyclodeaminase or a two‐step reaction involving the enzymes ornithine aminotransferase (OAT) and ProC (Figure 5). The pathway involving the cyclodeaminase was more common, being present in 169 isolates (pond: 77, soil: 92, Table 1), whereas the pathway variant involving the OAT was present in only 8 isolates (all pond).

For phenylalanine, there were two distinct pathways with a common first step involving a chorismate mutase generating prephenate. Subsequently, the pathway splits into two. In the first branch, prephenate is transformed into phenylpyruvate and finally to phenylalanine (enzymes: prephenate dehydratase, phenylpyruvate amino transferase (PPYAT)). In the second branch, prephenate is transformed into arogenate and then to phenylalanine (enzymes: prephenate amino transferase, arogenate dehydratase) (Figure 5). The pathway involving phenylpyruvate as an intermediate was more common occurring in 238 isolates (pond: 103, soil: 135, Table 1), whereas the pathway via arogenate occurred in 76 isolates (pond: 53, soil: 23).

### 2.6 Alternative Pathways Are Unevenly Distributed Across Habitats and Feature Strong Phylogenetic Signals

We then tested whether the specific alternative pathways were enriched in soil versus pond habitats. Using Fisher's exact test on contingency tables (Table 1), we found that alternative pathways were unevenly distributed between habitats (two‐sided Fisher's exact test, for methionine: *p* < 0.0001; for proline: *p* = 0.0065; for phenylalanine: *p* < 0.0001). For methionine, we found a significant enrichment of the B12‐dependent transsulfuration pathway among soil isolates and a corresponding enrichment of the B12‐dependent direct sulfhydrylation pathway among pond isolates (odds ratio, OR = 9.42, 95% CI: 3.2–37.7, Bonferroni‐corrected *p* < 0.0001). For proline, the ornithine amino transferase pathway exclusively occurred among pond isolates (OR = 0, 95% CI: 0–0.6, Bonferroni‐corrected *p* = 0.0102), whereas the other two pathways were more equally distributed among isolates of the two habitats (Table 1). For phenylalanine, there was an enrichment for the phenylpyruvate aminotransferase pathway among soil isolates and a corresponding enrichment of the arogenate dehydratase pathway among pond isolates (OR = 3, 95% CI: 1.7–5.5, *p* < 0.0001).

Next, we tested the strength of the phylogenetic signal for the alternative pathways. For this, we created a phylogenetic tree for our 314 natural isolates based on 1240 single copy orthogroups (Figures 6 and S7 for species prediction). We calculated the Fritz & Purvis' *D* statistics for each of the alternative pathways using binary (presence vs. absence) data (Fritz and Purvis 2010). The *D*‐value captures the phylogenetic signal of each pathway on a sliding scale from random (*D* ≥ 1) to intermediate (0 < *D* < 1) to strong (*D* ≤ 0). We found that alternative pathway distributions were strongly associated with phylogeny (*D*‐values significantly different from 1 indicated by *p*_random_, Table 1). For methionine, we found a strong phylogenetic signal for the B12‐dependent transsulfuration (*D* = 0.028, *p*_random_ < 0.0001) and for the B12‐dependent direct sulfhydrylation (*D* = −0.004, *p*_random_ < 0.0001), meaning that alternative pathway distribution is almost entirely determined by phylogeny. For proline, we found a similarly strong phylogenetic signal for all three alternative pathways (ornithine cyclodeaminase pathway: *D* = 0.023, *p*_random_ < 0.0001; standard proline biosynthesis pathways: *D* = 0.098, *p*_random_ < 0.0001; ornithine aminotransferase: *D* = 0.033, *p*_random_ < 0.0001). For phenylalanine, both pathways showed a fairly strong phylogenetic signal (both phenylpyruvate pathway and arogenate pathway: *D* = 0.198, *p*_random_ < 0.0001).

**FIGURE 6:**
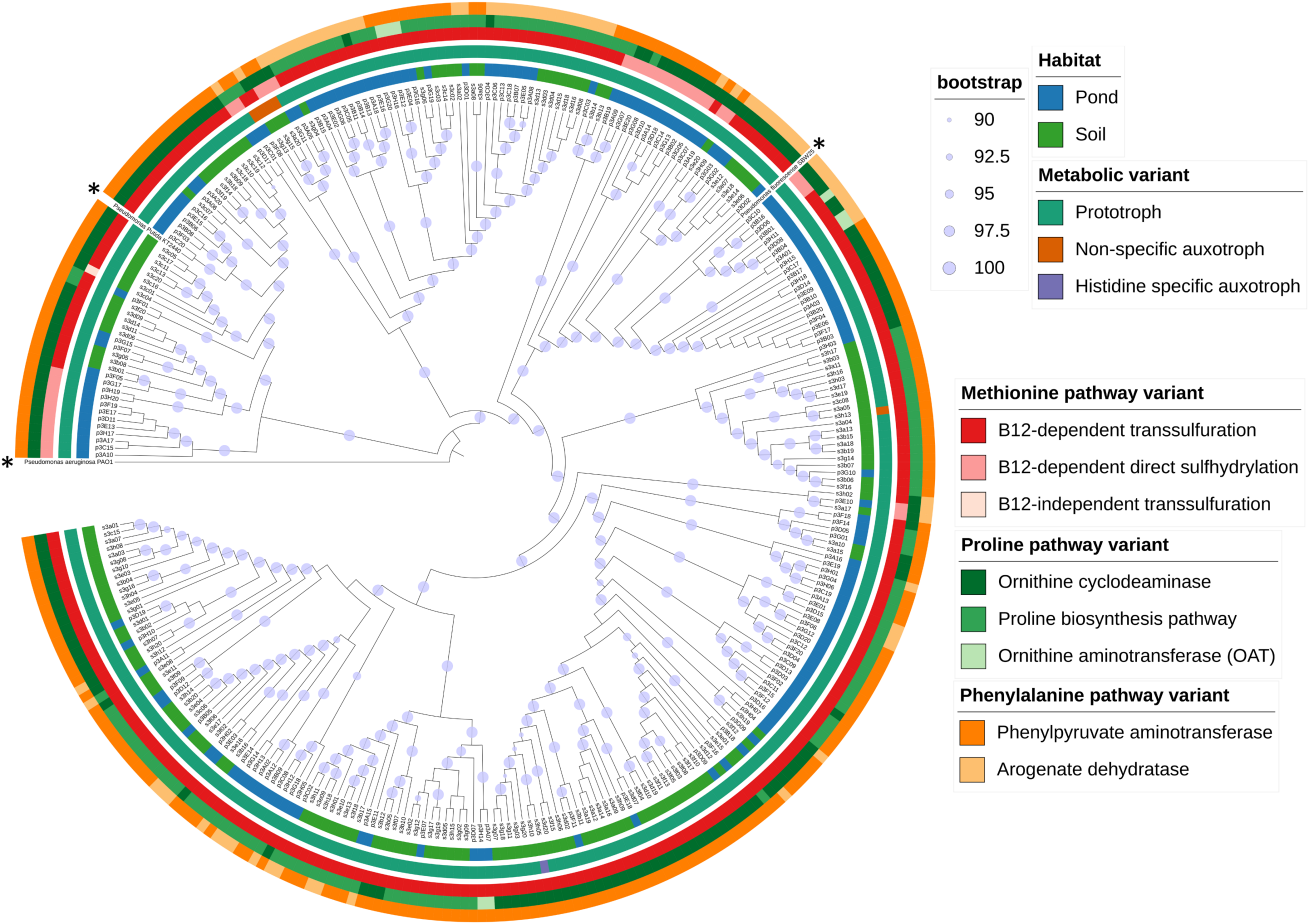
Strength of phylogenetic signal and habitat association vary across alternative amino acid biosynthetic pathways. The phylogeny of the 314 natural *Pseudomonas* isolates was assessed based on 1240 single copy orthogroups. *Pseudomonas aeruginosa* PAO1 was used as an outgroup, whereas *Pseudomonas putida* KT2440 and *Pseudomonas fluorescens* SBW25 were used as within‐tree references (denoted by asterisks). Blue circles (in various sizes) show bootstrap values from 90% to 100%. From inside to outside, coloured rings display the habitat of origin (soil vs. pond), the metabolic type (prototroph vs. auxotroph) and the alternative pathways for methionine, proline and phenylalanine. Alternative pathways show significant segregation according to phylogeny. The alternative pathways for phenylalanine show a significant habitat specific signal that is independent from phylogeny (see Table 1 for statistics).

To tease apart potential colinearities between habitat and phylogenetic signals, we used phylogenetic generalized linear mixed models for each alternative pathway by fitting habitat as a fixed effect and phylogenetic signal as a random effect. For methionine and proline, we found that any pattern of habitat‐specific enrichment disappeared when controlling for phylogeny (methionine B12‐dependent transsulfuration pathway: odds ratio, OR = 3.685, *p* = 0.1244; methionine B12‐dependent direct sulfhydrylation: OR = 0.212, *p* = 0.0907; proline ornithine cyclodeaminase pathway: OR = 2.033, *p* = 0.2233; proline biosynthesis pathway: OR = 0.753, *p* = 0.6231). Only for phenylalanine, we still recovered a habitat‐specific pathway enrichment after controlling for phylogenetic effects (phenylpyruvate aminotransferase pathway enriched among soil isolates and arogenate dehydratase pathway enriched among pond isolates: OR = 4.12, *p* = 0.0097). Overall, alternative pathways showed a strong phylogenetic association and therefore tend to be shared among closely related isolates. However, some alternative pathways like those for phenylalanine seem to preferentially occur in certain habitats.

## 3 Discussion

Metabolic auxotrophies, the inability to produce essential metabolites, are supposed to be common in microbial communities (Morris et al. 2012; Wolf and Koonin 2013; D'Souza et al. 2014; Zengler and Zaramela 2018; Johnson et al. 2020). The reason is that auxotrophy saves metabolic costs and thus generates fitness advantages as long as the essential metabolites can be acquired from the environment or from other community members overproducing and secreting them (D'Souza et al. 2018; Preussger et al. 2020; Kasalo et al. 2025). Auxotrophy can thus foster dependencies between microbial species and result in cross‐feeding interactions. However, the frequency of auxotrophy is often inferred from sequencing data using global databases (Price et al. 2020; Seif et al. 2020; Zimmermann et al. 2021; Ramoneda et al. 2023). Less clear is how common auxotrophies are among strains co‐isolated from the same community and how well in silico predictions match empirical data. Here, we combined experimental and bioinformatic approaches to detect amino acid auxotrophies in a collection of 315 natural *Pseudomonas* strains, co‐isolated from eight pond and eight soil communities. Our analyses revealed that experimental and bioinformatic approaches coincided in predicting high levels of prototrophy across habitats and communities. Moreover, both approaches reliably identified a single auxotrophic isolate for histidine. However, there were also several mismatches between the two approaches. While our experiments revealed one non‐specific auxotroph that was unable to grow in media without amino acids and four slow growers with fragile metabolic phenotypes, in silico approaches were unable to identify the basis of these dependencies. Conversely, one of the three bioinformatic pipelines used systematically overestimated auxotrophies. Taken together, we found low levels of amino acid auxotrophies (0.63%) among co‐isolated *Pseudomonas* strains from pond and soil communities and show that a careful integration of experimental and bioinformatic approaches is required to reliably assess specific and non‐specific auxotrophies.

At first sight, our findings contradict the perception that auxotrophies are ubiquitous in bacterial communities. However, a critical consideration is that the genus *Pseudomonas* is known for its metabolic and ecological versatility (Spiers et al. 2000; Silby et al. 2011). Given its ubiquity in many habitats and its generalist lifestyle, it is perhaps not surprising that the level of auxotrophy is low among *Pseudomonas* isolates (Ramoneda et al. 2023). It is important to note that we solely demonstrated low levels of auxotrophies for amino acids, whereas other auxotrophies like those for vitamins may be more common. However, since we used vitamin‐free media for all experiments, we are confident to conclude that auxotrophy levels are generally low among natural *Pseudomonas* isolates. Taken together, we propose that the debate should not be about whether levels of auxotrophies are generally high or low, but more about gaining a better understanding of the ecological conditions that either favour high versus low levels of auxotrophies. The evolution of auxotrophy and thus metabolic dependencies requires stable interactions between cross‐feeding partners. Stable interactions are more likely guaranteed when bacteria are adapted to specific niches and live in environments with little perturbations. Based on these considerations, we predict a negative correlation between auxotrophy frequency in a community and the proportion of taxa with a generalist (non‐niche specific) lifestyle. Similarly, we expect a negative correlation between auxotrophy frequency in a community and the frequency of environmental perturbations.

Bioinformatic approaches are powerful to predict auxotrophies and metabolic interactions from (meta‐) genome data and are especially useful to assess the metabolic potential of unculturable bacteria. However, our study shows that bioinformatic tools can both over and underestimate auxotrophies (see also Seif et al. 2020; Price et al. 2018). First, among the three different genome annotation tools used (Prokka, PGAP, ORFfinder), we found that Prokka tended to overestimate auxotrophy levels (Figure 4). The overestimation was predominantly associated with the non‐annotation of a single gene (*prs*), which is part of the histidine synthesis pathway. Previous research reported an open reading frame for *prs* in *P. fluorescens* with 67% nucleotide sequence similarity with *E. coli* K‐12 *prs* gene, but the annotation threshold used by Prokka is likely too stringent for the reliable annotation of this protein (Price et al. 2020). Second, we found that none of the bioinformatic pipelines could resolve the mechanistic basis of the non‐specific auxotrophy in p3F08 although such non‐specific auxotrophs seem to be quite common (D'Souza et al. 2014). Similarly, the bioinformatic pipelines could also not provide any mechanistic hints on the fragile slow growers. One reason might be that GapMind solely focuses on the actual amino acid synthesis pathways and not on co‐factors and vitamins that may also be required for optimal amino acid production. Accordingly, a lack of co‐factors could result in fragile slow‐growing phenotypes as observed for four isolates. Finally, previous bioinformatic work suggested that at least 40% of a synthesis pathway needs to be missing for reliably classifying a strain as auxotroph (Ramoneda et al. 2023). This contrasts with our results that reliably identified (through experiments and bioinformatics) a histidine auxotroph based on a single gene deletion. Thus, such stringent bioinformatic thresholds could underestimate auxotrophies and cause mismatches between bioinformatic and experimental data. These considerations show that an integration of empirical and bioinformatic approaches is a powerful approach to assess the frequency and mechanistic basis of bacterial auxotrophies.

Our bioinformatic analysis further identified the presence of alternative biosynthesis pathways among isolates for methionine, proline and phenylalanine. The distribution of these alternative pathways significantly correlated with the habitat type (pond vs. soil): strong association for methionine and phenylalanine; weaker association for proline. A strong habitat association could indicate that specific pathways are particularly beneficial in certain environments and may therefore be enriched in pond versus soil. In parallel, we observed strong phylogenetic signals for the distributions of all alternative amino acid pathways, suggesting that certain amino acid biosynthetic pathways became established in specific *Pseudomonas* lineages and stayed there conserved over evolutionary timescales. Importantly, we observed a positive collinearity between habitat effect and phylogenetic signal, and a phylogeny‐controlled analysis revealed that habitat‐specific enrichment of pathways only occurred for phenylalanine. Altogether, we observed strong phylogenetic signals for alternative pathway distributions and habitat‐specific pathway enrichment for certain amino acid pathways. It would thus be interesting to understand the ecological and evolutionary factors driving the divergence not only in *Pseudomonas* but also in other taxa.

In conclusion, we combined experimental with bioinformatic approaches to show that most natural *Pseudomonas* isolates from pond and soil habitats are amino acid prototrophs. The match between bioinformatic predictions and experimental results was robust overall, although there were certain bioinformatic pipelines that overestimated the frequency of auxotrophy and none of the pipelines could resolve the genetic basis of the non‐specific auxotroph and the four fragile slow‐growing isolates. While previous studies reported auxotrophy to be common in natural communities, we here show that it can be rare in certain taxa, like *Pseudomonas*. Our results contribute to the development of general ecological principles explaining variation in levels of auxotrophies across taxa and habitats. In particular, we propose that environmental conditions (stability versus perturbation) and lifestyle (specialist versus generalist) are key characteristics determining whether levels of auxotrophies and metabolic dependencies are high or low, respectively.

## 4 Material and Methods

### 4.1 Strain Collection

We used a collection of 315 natural *Pseudomonas* strains isolated from pond and soil samples collected on Irchel campus of the University of Zurich (47.39°N, 8.54°E), Switzerland (Butaitė et al. 2017, 2021). The initial sampling involved the collection of eight different soil and eight different pond samples, and the isolation of 20 *Pseudomonas* strains per sample. Among these 320 isolates, Sanger sequencing of the *rpoD* gene confirmed that 315 isolates belonged to the *Pseudomonas* genus and phylogenetic analyses show that most isolates cluster with either the *P. fluorescens* species complex, *P. putida* or *P. syringae* (Figure S7). Each isolate has an identification code, consisting of a habitat label (s = soil, p = pond), a location number (3 for Irchel campus), a community label (soil communities: lower case letters ‘a’ to ‘h’; pond communities: upper case letters ‘A’ to ‘H’) and an isolate number (1–20). We slightly modified the identification code compared to (Butaitė et al. 2018) by introducing the label ‘p’ for pond isolates.

As a prototrophic control strain for the main experiments, we used *Pseudomonas aeruginosa* PAO1. As auxotrophic control strains, we used a collection of 12 *Escherichia coli* K‐12 substr. BW25113 mutants from the KEIO collection (Baba et al. 2006), each featuring a transposon insertion in a specific amino acid synthesis gene, rendering them auxotrophs for the corresponding amino acid (Table S1). Additionally, to control that parental strains are prototrophic, we used WT *Escherichia coli* K‐12 substr. BW25113 and MG1665.

### 4.2 Media Preparation

For all experiments, we used M9 minimal medium supplemented with glucose (henceforth called M9G). Specifically, M9G consisted of pre‐prepared 1× M9 salts mix (KH_2_PO_4_ (3 g/L), NaCl (0.25 g/L), Na_2_HPO_4_ (6.78 g/L), NH_4_Cl (1 g/L)), MgSO_4_ × 7H_2_O (2 mM), CaCl_2_ × 2H_2_O (0.1 mM) and glucose (0.4%, m/v) as sole carbon sources. The M9 minimal medium was autoclaved while filter‐sterilized (0.2 μm) glucose and autoclaved MgSO_4_ × 7H_2_O and CaCl_2_ × 2H_2_O were added after autoclaving. For the initial screen of the 315 natural isolates, we prepared two variants of M9G in either liquid or solid (1.5% agar) form: M9G (i) without amino acids or (ii) supplemented with an undefined mixture of all amino acids through the addition of 2 g/L vitamin‐free casamino acid (CAA, casein hydrolysate). For the in‐depth screens with p3F08 and s3h05, we used the M9G agar medium and prepared additional variants supplemented with (iii) a defined mixture of all 20 amino acids, (iv) a single amino acid (SA = single addition) and (v) a mixture of 19 out of the 20 proteinogenic amino acids (SO = single omission). Finally, we also prepared M9G variants with double and triple amino acid additions and omissions for two sets of amino acids with interconnected synthesis pathways (isoleucine/leucine/valine and methionine/cysteine/serine). Aqueous amino acid stock solutions were prepared at a final concentration of 20 mM, filter‐sterilized (0.22 μm) and stored at 4°C for not more than 3 months. Individual amino acids were filter‐sterilized (0.2 μm) individually and consequently mixed and added to M9G to a final concentration of 50 μM. This concentration corresponds to the minimal concentration which resulted in distinct growth from the ‘no amino acid’ medium (see Figure S2). All above ingredients were purchased from Merck (Buchs SG, Switzerland) except for the individual amino acids, which were purchased from neoFroxx GmbH (Germany).

### 4.3 Preculture and Culture Conditions for the Auxotrophy Screen

We stored all isolates as glycerol stocks (25%) at −70°C. Before each screen, we revived the isolates by streaking them on Lysogeny broth (LB) agar plates, followed by incubation at 28°C for approximately 24 h. From each plate, we picked a single colony to inoculate liquid precultures consisting of 1.5 mL of M9G + CAA (see above), distributed in 24 well plates. Precultures were incubated at 28°C for 24 h and 170 RPM. Subsequently, they were washed twice with a 1× M9 salts solution followed by centrifugation at 9000 RPM for 2 min. The optical density (OD at 600 nm) of isolates was adjusted to 0.1. To prepare the inoculation of liquid culture, 96‐well plates (Eppendorf, non‐treated, flat bottom) were filled with 190 μL of liquid M9G versions (i) and (ii) and 10 μL of OD‐adjusted precultures. The plates were subsequently incubated at 28°C and 170 rpm in a shaking incubator (Infors HT, Multitron Standard Shaker). The OD_600_ was measured after 0 and 24 h with a microplate reader (Tecan, Infinite MPlex). The relative growth value was then calculated by dividing the OD of each isolate in the medium without amino acids to the one in the medium with CAA after 24 h.

To prepare the experimental agar plates, we filled large round culture dishes (150 × 20 mm, Sarstedt, Germany) with 50 mL of hot M9G agar. The version of the M9G agar used (i)–(v) depended on the screen with the amino acid mixture being added after autoclaving as soon as the media was cooled down to approximately 60°C. For the initial auxotrophy screen with all 315 isolates, we used M9G agar versions (i) and (ii). For the in‐depth screening of the putative auxotrophs, we used M9G agar versions (i) and (iii)–(v). For each screen, we spotted 2 μL droplets of each OD‐adjusted isolate using a Viaflo 96 automated pipetting system (Integra Biosciences, Switzerland). Each plate was seeded with triplicates of 28 environmental isolates, *P. aeruginosa* PAO1 (prototroph control), *E. coli* *ΔilvA* and *E. coli ΔmetA* (auxotroph control) and six medium blanks to check for contamination. Droplets were dried for 10 min under the hood and plates were then incubated at 28°C for 24 h. Subsequently, we imaged each agar plate using a FUSION FX6 EDGE Imaging System (Witec, Switzerland) and qualitatively assessed colony growth by comparing growth between media with and without (all or specific) amino acids. Specifically, colony growth on media containing all amino acids was taken as the standard for each isolate. Then, we compared this standard to colony growth of the corresponding isolate on media without (all or specific) amino acids. We defined three categories relative to the reference standard: equal growth, reduced growth and no growth. For all the isolates that showed reduced growth, we repeated the experiment and incubated them for 48 h to confirm that reduced growth is due to auxotrophy and not simply due to slow growth.

For a subset of experiments (mainly for the data shown in Figure 3), we used Fiji (ImageJ, version 2.16.0/1.54p) (Schindelin et al. 2012) to quantitatively assess colony growth. Specifically, we manually selected the area of interest (ROI, i.e., the entire colony on the plate) and measured the average pixel intensity of the ROI. For each colony, we subtracted the average pixel intensity of the agar background from the ROI pixel intensity. Finally, we calculated the relative growth as the average pixel intensity of the isolate in the medium of interest divided by the average pixel intensity of the same isolate in the medium supplemented with all amino acids.

### 4.4 Whole Genome Sequencing and GapMind Analysis

We sequenced the whole genomes of all 315 isolated using Illumina short read sequencing technology. A subset of 24 isolates was sequenced as part of a separate study using Illumina Hiseq 2500 (2 × 250 bp paired end) and Shovill for genome assembly (a SPAdes based assembler)(Butaitė et al. 2017). The remaining isolates were sequenced in multiple batches by MicrobesNG (Birmingham, UK, https://microbesng.com/) using illumina NovaSeq 6000 (Illumina, San Diego, USA) (2 × 250 bp paired end, with 30× coverage). Here is a brief extract of the protocol used by Microbes NG. The genomic DNA libraries were prepared by MicrobesNG using a Nextera XT Library Prep Kit (Illumina, San Diego, USA) following manufacturer's protocol with a modification to the DNA input (twofold increase) and a PCR step (elongation time increased to 45 s). Pipetting for DNA quantification and library preparation were done using a Hamilton Microlab STAR automated liquid handling system (Hamilton, Bonaduz AG, Switzerland). Adapter sequences were trimmed from reads using Trimmomatic (version 0.30) with a quality cutoff of Q15. Quality controlled was performed by MicrobesNG's in‐house pipelines (Samtools, BedTools and bwa‐mem). De novo genome assembly was also performed by MicrobesNG using SPAdes (version 3.7). Using these assembled genomes, we performed the genome annotation with three different tools: Prokka 1.14.3, NCBI PGAP 2024‐04‐27.build7426 (Li et al. 2021) specifying *Pseudomonas* sp. as the organism and NCBI ORFfinder 0.4.3 (with genetic code 11 and default settings), which is not biased by homology‐based gene prediction. We then used GapMind (Price et al. 2020) with PaperBLAST commit 93e68cf (Price and Arkin 2017) to predict amino acid biosynthesis pathways and gaps therein, separately for each of the three annotations. We developed a Python script to summarize auxotrophies, whereas plots and data analysis were done with R (version = 4.4.3).

### 4.5 Phylogenetic Analysis

We assessed the orthology inference with OrthoFinder (Emms and Kelly 2019) and further used the single copy orthogroups to reconstruct the phylogenetic association among the 314 sequenced *Pseudomonas* isolates. For orthology inference, we integrated three reference strains into our phylogenetic analysis: *P. aeruginosa* PAO1 (RefSeq: NC_002516.2) as an outgroup and *P. fluorescens* SBW25 (RefSeq: NC_012660.1) and *P. putida* KT2440 (RefSeq: NZ_CP169744.1) as intra‐tree references. We used all the identified single copy orthologue sequences (*n* = 1240) to create the phylogenetic tree. In detail, these 1240 protein sequences were aligned using MAFFT (opt: ‐‐auto) and trimmed with trimAI (opt: ‐automated1). The trimmed aligned sequences were concatenated using a python script. Finally, IQ‐TREE2 was used on the concatenated sequence with following parameter settings: ‐m LG + R7, ‐bb 1000, ‐alrt 1000, ‐nt 16. IQ‐TREE2 ran on 317 sequences of 386,854 aligned amino acids among which there were: 146,841 distinct patterns, 145,116 parsimony‐informative, 26,454 singleton sites, 215,284 constant sites. To visualize the generated tree file, we used the tree visualization web platform iTOL (Letunic and Bork 2024). To annotate the phylogenetic tree, we generated iTOL datasets using R.

### 4.6 Statistical Analysis

To assess whether pathways are differentially distributed across habitats, we first performed Fisher's exact test on contingency tables [*h* × *n*] with ‘*h*’ the number of habitats = ‘2’ and ‘*n*’ the number of alternative pathways per amino acid (*n* = 2 for phenylalanine and *n* = 3 for methionine and proline). For methionine and proline, where contingency tables were 2x3, we computed *p*‐values with 10,000 Monte Carlo simulations as described in the R package {stats}. We then performed post hoc Fisher's exact tests for each of the alternative pathways (per amino acid) by comparing its frequency in pond and soil relative to the frequencies of the sum of the other pathways (2 × 2 contingency tables). We corrected *p*‐values for multiple testing by using the Bonferroni approach.

We used Fritz and Purvis' *D* statistics to quantify the phylogenetic signal of each alternative pathway. We considered alternative pathway abundance as a binary variable (present or absent). Statistical tests comprised 10,000 permutations and *D*‐value < 1 with *p*_rand_ < 0.05 to reject the null hypothesis of random trait distribution. We used the Bonferroni approach to correct *p*‐values for multiple testing. No statistical tests were performed for the alternative methionine pathway that occurred only once.

To disentangle phylogenetic signals of alternative pathways from habitat‐specific distributions, we ran a phylogenetic generalized linear mixed model (pglmm from the R package ‘phyr’) (Li et al. 2020) by fitting the binary (presence/absence) state of alternative pathways for each isolate as a response, the habitat (pond or soil) as a fixed factor and the phylogenetic covariance matrix calculated from the phylogenetic tree as a random factor (Figures 6 and S5). The reported odds ratios (OR) are the exp(*ß*) values of the fixed effect.

## Author Contributions

**Simon Maréchal:** conceptualization, methodology, data curation, investigation, visualization, writing – original draft, writing – review and editing, software, formal analysis. **Benjamin Heiniger:** software, data curation, formal analysis, writing – review and editing. **Shaohua Gu:** software, data curation, formal analysis, writing – review and editing. **Swagatika Dash:** methodology, writing – review and editing. **Christian H. Ahrens:** writing – review and editing, supervision. **Rolf Kümmerli:** supervision, writing – review and editing, conceptualization, investigation, validation, funding acquisition, project administration, resources, writing – original draft.

## Funding

This work was supported by Schweizerischer Nationalfonds zur Förderung der Wissenschaftlichen Forschung (212266, 197391).

## Conflicts of Interest

The authors declare no conflicts of interest.

## Supporting information


**Table S1:** emi70434‐sup‐0001‐Supinfo.docx. *E. coli* K12 mutants from the KEIO collection used in this study with a description of the mutated gene and its role in the respective amino acid biosynthesis pathway (from EcoCyc database). The mutants were previously described in Mee et al. (2014). Genome Biology 20: 238.
**Figure S1:** Results from amino acid growth assays of *Pseudomonas* isolates on M9G agar and in M9G liquid medium are strongly correlated. The 315 *Pseudomonas* isolates were grown in liquid or on agar‐based M9G medium supplemented with either all or none of the proteinogenic amino acid. The relative growth on agar was assessed qualitatively and categorized as described in the legend of Figure 1. The relative growth in liquid medium was assessed quantitatively: OD_600_‐value measured in M9G without amino acids divided by OD_600_‐value measured in M9G with all amino acids. The correlation between growth in both media was significant (Spearman's rank‐order correlation; Rho = 0.22, *p* < 0.0001).
**Figure S2:** Representative picture from a preliminary screen to assess the minimal concentration of each of the 20 proteinogenic amino acids required to allow auxotrophs to grow. To assess the optimal concentration of amino acids required to allow auxotrophs to grow, we prepared M9G medium with no amino acids and a series of M9G media with all amino acids but differing in their final concentration, ranging from 10, 50, 100, 150 to 200 μM (from top row to bottom row). We tested the growth of (from left to right): an auxotrophic control *E. coli* ∆proA, a prototrophic control *P. aeruginosa* PAO1, two auxotrophic isolates (s3h05 and p3F08), one slow growing isolate (s3g13) and a prototrophic isolate (p3F14). We finally decided to use 50 μM (per amino acid) for the main experiments as it was the minimal concentration required to visibly promote the growth of auxotrophs.
**Figure S3:** Validation of the single amino acid omission (SO) and addition (SA) approach to screen for auxotrophy and exploring the valine‐induced isoleucine starvation in *E. coli* . (A) The prototrophic PAO1 and 12 auxotrophic *E. coli* from the KEIO collection were used to validate our experimental approach. Each strain was grown in M9G agar with all amino acids and in M9G media supplemented with 19 out of the 20 proteinogenic amino acids (SO = single omission). See material and method for exact media composition. Data show qualitative growth of strains under SO conditions relative to M9G with all amino acids. (B) The prototrophic PAO1 and two auxotrophic *E. coli* from the KEIO collection ( *E. coli* ∆ilvA and *E. coli* ∆metA) were grown in M9G with all amino acids and M9G supplemented with a single amino acid (SA = single addition). Data show growth of strains under SA conditions relative to M9G with all amino acids. Results from SO and SA experiments show that specific auxotrophies can reliably be detected with our approach. However, the results also revealed an anomaly, namely that none of the *E. coli* strains could grow when isoleucine was absent in the medium. We suspected this anomaly to be based on the so‐called valine‐induced isoleucine starvation, meaning that the presence of valine suppresses isoleucine synthesis even in prototrophs. (C) Experiments with two *E. coli* wild type strains (BW25113 and MG1665) confirmed the absence of colony growth when isoleucine was absent, although both strains are prototrophs. However, we observed the appearance of small colonies after 48 h growth in M9G without isoleucine (SO). We suspected these colonies to be mutants that circumvented valine‐induced isoleucine starvation. We picked one of these colonies and henceforth named it BW25113 valine‐resistant mutant. (D) Experiments with the BW25113 valine‐resistant mutant yielded normal growth patterns for this isolate under all amino acid SA/SO conditions, even in the absence of isoleucine, thus confirming that the anomaly in the wild type strains is explained by the valine‐induced isoleucine starvation phenomenon.
**Figure S4:** Comparing qualitative manual with quantitative Fiji‐based scoring of colony growth for the isolate p3F08. The growth phenotype of p3F08 is visually difficult to assess due to the so‐called coffee ring effect. This effect suggests growth at the rim of the colony but not inside. The strength of the rim varies across amino acid compositions of the medium, and it is unclear whether the rim is the result of bacterial growth or an artefact. The figure shows representative examples of colony growth after 48 h across a range of single amino acid addition (SA) conditions. A comparison of qualitative vs. quantitative growth assessments revealed that growth tends to be overestimated with the qualitative manual scoring. For this reason, we solely used the quantitative Fiji scoring for the analysis presented in Figure 3.
**Figure S5:** Manual qualitative colony growth scores correlate with quantitative Fiji scores. To confirm that the qualitative manual scoring of colonies used throughout the manuscript is an adequate method, we compared our qualitative growth scores to quantitative growth scores obtained with Fiji‐based image analysis. We conducted this experiment with a subset of natural isolates, control strains and conditions (mainly for the results shown in Figures 3 and S3D) and found strong correlations between the two scoring methods (Spearman's rank‐order correlation after 24 h: Rho = 0.85, *p* < 0.00001; after 48 h: Rho = 0.73, *p* < 0.00001; *N* = 582).
**Figure S6:** Detailed GapMind predictions of amino acid biosynthesis pathway based on Prokka annotation. Biosynthetic pathways for 17 amino acids predicted by GapMind for 314 natural Pseudomonas isolates: (A) Arginine, Asparagine, Cysteine, Glutamine and Glycine; (B) Histidine, Isoleucine, Leucine, Lysine, Methionine and Phenylalanine; (C) Proline, Serine, Threonine Tryptophan, Tyrosine, Valine. Natural isolates (*y*‐axis) in each panel are separated into four categories based on experimental results (from top to bottom: histidine specific auxotroph, non‐specific auxotroph, slow growers, prototrophs). Each amino acid biosynthetic pathway is broken down into a series of genes (*x*‐axis) forming a complete biosynthetic pathway. When a pathway is detected by Gapmind, it assigns a colour representing the confidence level with which every gene involved in the detected pathway are found (green = high confidence, red = low confidence, yellow = intermediate confidence). When a pathway is detected but an essential gene is missing (below low confidence), it will be labelled as ‘Expected but not identified’ (black). When a pathway is not detected, individual genes will not be searched by GapMind and will be labelled as ‘Not expected’ (grey).
**Figure S7:** Phylogenetic tree of the 314 natural Pseudomonas isolate with main represented species groups. Phylogeny was constructed using 1240 single copy orthogroups (from Orthofinder). Allocation to species groups was determined using Kraken using whole genome data and was carried out by MicrobesNG. Note that Kraken taxonomic allocation comes with substantial uncertainty. While the affiliation of isolates to the Pseudomonas genus occurred with high confidence (91% ± 6%), the species affiliation was associated with much lower confidence (52% ± 28%).

Data S1.

## Data Availability

The data that supports the findings of this study are available in the Supporting Information of this article. Raw sequencing data are publicly available on European Nucleotide Archive under the study numbers “PRJEB127116” and “PRJEB76792”.
