# Supplementary material for: Low Levels of Amino Acid Auxotrophy Among Environmental Pseudomonas Isolates
Source: Environ Microbiol. 2026 Oct 2;28(10):e70434. doi: 10.1111/1462-2920.70434 (PMC13633446; doi:10.1111/1462-2920.70434)
Supplement: Supplementary file 1 [file EMI-28-e70434-s002.docx]

**Low levels of amino acid auxotrophy among environmental *Pseudomonas* isolates**

Simon Maréchal^1^, Benjamin Heiniger^2,3^, Shaohua Gu^4^, Swagatika Dash^5^, Christian H. Ahrens^2,6^, Rolf Kümmerli*^1^

^1^ Department of Quantitative Biomedicine, University of Zürich, Switzerland

^2^ Method Development and Analytics, Agroscope, Zürich, Switzerland

^3^ PhD Program Systems Biology, Zurich Life Sciences Graduate School

^4^ College of Resources and Environmental Sciences, State Key Laboratory of Nutrient Use and Management (SKL-NUM), National Academy of Agriculture Green Development, China Agricultural University, Beijing 100193, China

^5^ Department of Ecology, School of Biology/Chemistry, University of Osnabrück, Osnabrück, Germany

^6^ Swiss Institute of Bioinformatics

*Correspondence: Rolf Kümmerli, [rolf.kuemmerli@uzh.ch](mailto:rolf.kuemmerli@uzh.ch)

**This file contains:**

- 1 Supplementary Table
- 7 Supplementary Figures

**Supplementary Tables**

**Table S1.** *E.coli* K12 mutants from the KEIO collection used in this study with a description of the mutated gene and its role in the respective amino acid biosynthesis pathway (from EcoCyc database). The mutants were previously described in Mee et al. 2019. Genome Biol 20: 238.

| **Gene name** | **Gene function (extracted from**  [**https://ecocyc.org/ECOLI/NEW-IMAGE?type=PATHWAY&object=Amino-Acid-Biosynthesis**](https://ecocyc.org/ECOLI/NEW-IMAGE?type=PATHWAY&object=Amino-Acid-Biosynthesis) |
| --- | --- |
| *argA* | N-acetylglutamate synthase *(*ArgA) carries out the first steps in the superpathway of arginine biosynthesis and the pathway of ornithine biosynthesis. |
| *cysE* | Serine acetyltransferase carries out the first step in the pathway of cysteine biosynthesis, converting L-serine into O-acetyl-L-serine. |
| *hisB* | HisB catalyzes two key reactions in histidine biosynthesis. |
| *ilvA* | Threonine deaminase (IlvA) carries out the first step in the synthesis of isoleucine. |
| *leuB* | catalyzes the first (LeuA)/third (LeuB) and second (LeuC) committed step in leucine biosynthesis |
| *lysA* | Diaminopimelate decarboxylase (LysA) catalyzes the last step in lysine biosynthesis |
| *metA* | (MetA) catalyzes the first unique step in the de novo methionine biosynthesis pathway in Escherichia coli |
| *pheA* | (PheA) carries out the shared first step in the parallel biosynthetic pathways for the aromatic amino acids tyrosine and phenylalanine, as well as the second step in phenylalanine biosynthesis. |
| *proA* | (ProA) catalyzes the the reduction of γ-glutamyl phosphate to glutamate 5-semialdehyde, the second reaction in the L-proline biosynthesis I (from L-glutamate) pathway |
| *thrC* | Threonine synthase (ThrC) carries out the final step in the biosynthesis of L-threonine |
| *trpC* | Bifunctional phosphoribosylanthranilate isomerase / indole-3-glycerol phosphate synthase (TrpC) carries out the third and fourth steps in the tryptophan biosynthesis pathway. |
| *tyrA* | Bifunctional chorismate mutase / prephenate dehydrogenase (TyrA) carries out the shared first step in the parallel biosynthetic pathways for the aromatic amino acids tyrosine and phenylalanine, as well as the second step in tyrosine biosynthesis. |

**Supplementary Figures**

**Figure S1. Results from amino acid growth assays of *Pseudomonas* isolates on M9G agar and in M9G liquid medium are strongly correlated.** The 315 *Pseudomonas* isolates were grown in liquid or on agar-based M9G medium supplemented with either all or none of the proteinogenic amino acid. The relative growth on agar was assessed qualitatively and categorized as described in the legend of Figure 1. The relative growth in liquid medium was assessed quantitatively: OD_600_-value measured in M9G without amino acids divided by OD_600_-value measured in M9G with all amino acids. The correlation between growth in both media was significant (Spearman’s rank-order correlation; Rho = 0.22, p < 0.0001)

**Figure S2. Representative picture from a preliminary screen to assess the minimal concentration of each of the 20 proteinogenic amino acids required to allow auxotrophs to grow.** To assess the optimal concentration of amino acids required to allow auxotrophs to grow, we prepared M9G medium with no amino acids and a series of M9G media with all amino acids but differing in their final concentration, ranging from 10, 50, 100, 150 to 200µM (from top row to bottom row). We tested the growth of (from left to right): an auxotrophic control E. coli ∆proA, a prototrophic control P. aeruginosa PAO1, two auxotrophic isolates (s3h05 and p3F08), one slow growing isolate (s3g13) and a prototrophic isolate (p3F14). We finally decided to use 50µM (per amino acid) for the main experiments as it was the minimal concentration required to visibly promote the growth of auxotrophs.

**Figure S3.** **Validation of the single amino acid omission (SO) and addition (SA) approach to screen for auxotrophy and exploring the valine-induced isoleucine starvation in E. coli**. **A)** The prototrophic PAO1 and 12 auxotrophic E. coli from the KEIO collection were used to validate our experimental approach. Each strain was grown in M9G agar with all amino acids and in M9G media supplemented with 19 out of the 20 proteinogenic amino acids (SO = single omission). See material and method for exact media composition. Data show qualitative growth of strains under SO conditions relative to M9G with all amino acids. **B)** The prototrophic PAO1 and two auxotrophic E. coli from the KEIO collection (E. coli ∆ilvA and E. coli ∆metA) were grown in M9G with all amino acids and M9G supplemented with a single amino acid (SA = single addition). Data show growth of strains under SA conditions relative to M9G with all amino acids. Results from SO and SA experiments show that specific auxotrophies can reliably be detected with our approach. However, the results also revealed an anomaly, namely that none of the E. coli strains could grow when isoleucine was absent in the medium. We suspected this anomaly to be based on the so-called valine-induced isoleucine starvation, meaning that the presence of valine suppresses isoleucine synthesis even in prototrophs. **C)** Experiments with two E. coli wild type strains (BW25113 and MG1665) confirmed the absence of colony growth when isoleucine was absent, although both strains are prototrophs. However, we observed the appearance of small colonies after 48h growth in M9G without isoleucine (SO). We suspected these colonies to be mutants that circumvented valine-induced isoleucine starvation. We picked one of these colonies and henceforth named it BW25113 valine-resistant mutant. **D)** Experiments with the BW25113 valine-resistant mutant yielded normal growth patterns for this isolate under all amino acid SA/SO conditions, even in the absence of isoleucine, thus confirming that the anomaly in the wild type strains is explained by the valine-induced isoleucine starvation phenomenon.

**Figure S4.** **Comparing qualitative manual with quantitative Fiji-based scoring of colony growth for the isolate** **p3F08.** The growth phenotype of p3F08 is visually difficult to assess due to the so-called coffee ring effect. This effect suggests growth at the rim of the colony but not inside. The strength of the rim varies across amino acid compositions of the medium, and it is unclear whether the rim is the result of bacterial growth or an artifact. The figure shows representative examples of colony growth after 48h across a range of single amino acid addition (SA) conditions. A comparison of qualitative vs. quantitative growth assessments revealed that growth tends to be overestimated with the qualitative manual scoring. For this reason, we solely used the quantitative Fiji scoring for the analysis presented in Fig. 3.

**Figure S5.** **Manual qualitative colony growth scores correlate with quantitative Fiji scores**. To confirm that the qualitative manual scoring of colonies used throughout the manuscript is an adequate method, we compared our qualitative growth scores to quantitative growth scores obtained with Fiji-based image analysis. We conducted this experiment with a subset of natural isolates, control strains, and conditions (mainly for the results shown in Fig. 3 and Fig. S3D) and found strong correlations between the two scoring methods (Spearman’s rank-order correlation after 24h: Rho = 0.85, p < 0.00001; after 48h: Rho = 0.73, p < 0.00001; N = 582).

**Figure S6. Detailed GapMind predictions of amino acid biosynthesis pathway based on Prokka annotation.** Biosynthetic pathways for 17 amino acids predicted by GapMind for 314 natural Pseudomonas isolates: **A)** Arginine, Asparagine, Cysteine, Glutamine and Glycine; **B)** Histidine, Isoleucine, Leucine, Lysine, Methionine and Phenylalanine; **C)** Proline, Serine, Threonine Tryptophan, Tyrosine, Valine. Natural isolates (y axis) in each panel are separated into four categories based on experimental results (from top to bottom: histidine specific auxotroph, non-specific auxotroph, slow growers, prototrophs). Each amino acid biosynthetic pathway is broken down into a series of genes (x-axis) forming a complete biosynthetic pathway. When a pathway is detected by Gapmind, it assigns a color representing the confidence level with which every gene involved in the detected pathway are found (green = high confidence, red = low confidence, yellow = intermediate confidence). When a pathway is detected but an essential gene is missing (below low confidence), it will be labeled as “Expected but not identified” (black). When a pathway is not detected, individual genes will not be searched by GapMind and will be labeled as “Not expected” (grey).

**Figure S7. Phylogenetic tree of the 314 natural Pseudomonas isolate with main represented species groups.** Phylogeny was constructed using 1240 single copy orthogroups (from Orthofinder). Allocation to species groups was determined using Kraken using whole genome data and was carried out by MicrobesNG. Note that Kraken taxonomic allocation comes with substantial uncertainty. While the affiliation of isolates to the Pseudomonas genus occurred with high confidence (91% ± 6%), the species affiliation was associated with much lower confidence (52% ± 28%).
